# Real‐World Practice Patterns in Diagnosis and First‐Line Treatment in Metastatic Breast Cancer

**DOI:** 10.1155/tbj/4333748

**Published:** 2026-06-28

**Authors:** Poorni M. Manohar, Veena Shankaran, Nancy E. Davidson, Natasha Hunter, William R. Gwin, Rachel L. Yung, Jennifer M. Specht, Catherine Federenko, Sun Qin, Qian (Vicky) Wu, Jenna M. Voustinas, Josh A. Roth, Hannah M. Linden

**Affiliations:** ^1^ Department of Internal Medicine, Rush University Medical Center, Chicago, Illinois, USA, rush.edu; ^2^ Department of Internal Medicine, University of Washington/Fred Hutchinson Cancer Research Center, Seattle, Washington, USA; ^3^ Department of Internal Medicine, Fred Hutchinson Cancer Research Center, Seattle, Washington, USA, fredhutch.org; ^4^ Pfizer Inc., New York, New York, USA, pfizer.com; ^5^ CHOICE Institute, University of Washington, Seattle, Washington, USA, washington.edu

**Keywords:** biopsy, CDK4/6 inhibitors, metastatic breast cancer, quality of care, treatment patterns

## Abstract

**Intro:**

Divergence from national guidelines and variations in practice patterns impact care and outcomes in patients with metastatic breast cancer (MBC). We sought to assess the quality of care in the diagnosis and treatment of real‐world patients with MBC in Washington State.

**Methods:**

Data were retrospectively analyzed using a linked cancer registry and insurance claims platform for patients with recurrent or de novo MBC diagnosed between 2008 and 2019.

**Results:**

We identified 1101 patients with MBC (median age: 66), 715 recurrent and 386 de novo. Most patients were White (89%), all were insured (Commercial [47%], Medicaid [4%], Medicare [35%], or multiple [13%]), and 15% lived in areas of high deprivation (Area Deprivation Index [ADI]: 8–10). Of the patients with recurrent MBC, less than half received a biopsy (49.5%) or biomarker reassessment (48.7%) to confirm the diagnosis of MBC. Patients treated at high‐ and medium‐volume centers had higher rates of biopsy than low‐volume clinics (51.9%, 54.3%, and 40.7%, respectively, *p* = 0.03). ET alone was more common in patients who did not undergo biopsy (62.3% vs. 37.7%, *p* < 0.001) or biomarker reassessment (62.7% vs. 37.3%, *p* < 0.001). Among the 677 patients with estrogen receptor (ER)+/HER2− MBC (de novo and recurrent), most received ET alone (69%), followed by CT (22%) and CDKi + ET (9%). Importantly, 40% of patients were treated before CDK4/6i approval. Most patients who received CDKi + ET were < 65 years old (65.2%, *p* < 0.02). Patients with commercial insurance were more likely to receive CDKi + ET compared to those with Medicare/Medicaid. (60.9% vs. 26.1%, *p* = 0.10).

**Conclusion:**

Our findings highlight key gaps in MBC management and serve as a launch point for patient‐centered and quality‐promoting initiatives.

## 1. Introduction

Although breast cancer outcomes have improved due to therapeutic advances, 20% of patients diagnosed with breast cancer will present with or develop metastatic disease [1, 2]. Because receptor status can change between the primary tumor and metastatic disease—with reported discordance rates approaching 20%—professional societies advocate tissue confirmation and repeat receptor testing at recurrence (Level 1 evidence) [3–6]. When reassessment is omitted, clinicians may inadvertently base treatment decisions on outdated or inaccurate tumor biology [6–8]. Population‐level evidence describing how often these diagnostic steps occur in routine practice remains limited, particularly outside academic trial settings.

Despite broad consensus on available therapeutic classes, the relative timing of endocrine therapy (ET), targeted agents, and chemotherapy (CT) in metastatic breast cancer (MBC) often remains individualized in practice [4, 9–12]. Prescribing patterns can be based on the experience and comfort level of providers [13]. Furthermore, widespread implementation of novel therapies can lag by up to a decade, despite incorporation into national guidelines and approval by the FDA [14–16]. The lack of clear recommendations for treatment sequencing, combined with known delays in the incorporation of novel therapies into practice, may contribute to CT overutilization, which negatively impacts patients’ quality of life compared to targeted agents [17–19]. Improving rates of survival, with 5‐year survival rates nearing 40% in the United States, for patients with MBC necessitates the use of evidence‐based care to ensure the best outcomes [20].

We hypothesized that adherence to national guidelines may not be reflected in real‐world settings because of the complexities of diagnosis and treatment selection in MBC. Our objective is to examine real‐world practice patterns in the management of MBC and explore predictors of nonadherence. Cost analysis will highlight the financial impact on health systems and patients. Population‐based analyses such as ours can help identify specific clinical settings and patient groups where guideline‐concordant care may be less consistently delivered.

## 2. Methods

### 2.1. Database

We utilized the Hutchinson Institute for Cancer Outcomes Research (HICOR) database, which links clinical and survival data from CSS‐Puget Sound Surveillance Epidemiology and End Results (SEER) and the Washington State Cancer Registry with enrollment and claims data from two large regional commercial insurers (Premera Blue Cross and Regence Blue Shield), Medicare and Medicaid. Collectively, these data sources capture approximately 80% of cancer cases in Washington. The study was approved by the University of Washington Ethics Committee/IRB.

### 2.2. Cohort Selection

We identified two cohorts: patients with recurrent MBC and patients with de novo MBC. To identify recurrent MBC, we first identified all patients with primary breast cancer (Stage I–III) diagnosed between 2008 and 2019 in the HICOR database. Patients were categorized with a distant recurrence if they met one of three definitions: (1) ICD 9/10 codes for metastatic disease, (2) resumption of systemic therapy (CT or radiation) after a minimum gap of 4 months from completion of therapy for early breast cancer, or (3) resumption of ET after a minimum gap of 12 months from completion of therapy for early breast cancer. De novo MBC (Stage IV) patients were identified directly through the state cancer registry. The date of diagnosis of recurrent or de novo MBC was delineated as the index date (time 0). Patients were required to be continuously enrolled in one of the insurance plans from the date of diagnosis of primary breast cancer through the index date (date of recurrence) plus 12 months or from the date of diagnosis of de novo MBC through 12 months. Patients with diagnosis codes for a secondary malignancy were excluded. To minimize capturing local recurrences, patients with breast cancer surgery 12 months after the index date were excluded.

### 2.3. Biopsy and Biomarker Testing

Patients with recurrent MBC were assessed for receipt of biopsy and biomarker reassessment (estrogen receptor [ER]/PR and HER2) at the time of recurrence. Billing codes in insurance claims for biopsies, immunohistochemistry stains, and fluorescence in situ hybridization testing were identified after recurrent MBC diagnosis. A 3‐month window was allowed to account for delays in billing claims and capture an accurate pattern of biopsy.

### 2.4. First‐Line Selection

Treatment selection within 6 months of MBC diagnosis was evaluated in the ER+/HER2− MBC cohort (comprised of recurrent MBC and de novo patients). Therapies provided after the date of MBC diagnosis were identified through the use of all diagnoses, procedures, and J codes reflecting receipt of specific drugs. Treatment was categorized as ET alone, CDK4/6 inhibitors with ET (CDKi + ET), or CT.

### 2.5. Cost Assessment

We estimated costs from the payee (patient) and payor (insurance) perspectives for 1‐year postdiagnosis of MBC. Payee cost responsibility was defined as payment to providers for which beneficiaries were responsible through deductibles, coinsurance, and copayments, in the year after the index diagnosis date. Payor cost responsibility was calculated from cumulative reimbursement amounts derived from claims in the year after the index diagnosis date. Overall and healthcare service–stratified costs to beneficiaries were identified. The information extracted from the database was categorized as inpatient costs, outpatient costs, and outpatient pharmacy costs.

### 2.6. Factors Influencing Outcomes

We examined patient, disease, and institutional factors that may influence outcomes. Patient data include age, sex, modified Charlson Comorbidity Index (comorbidity), race, Area Deprivation Index (ADI), neighborhood of residence, and insurance (Commercial, Medicare, Medicaid, or multiple). The ADI considers socioeconomic status, housing cost, and quality and material deprivation. ADI is a more sensitive measure of socioeconomic status and is calibrated specifically to Washington state rather than national disparities scores [21]. We attributed patients to the clinic site most likely to direct the majority of their cancer care during the period of interest. Institutions are classified as rural or urban using the Rural Urban Commuting Area (RUCA) classification. Clinics are identified using Tax ID numbers (TINs) or CMS Certification Numbers (CCNs) on health insurance claims. Clinics were further classified by the number of breast cancer patients treated annually at the center: high volume (> 100 patients), medium volume (> 25 and < 100 patients), and low volume (< 25 patients).

### 2.7. Statistical Analysis

Medians and ranges (min–max) were calculated for continuous variables, and counts and percentages were calculated for categorical variables. For group comparisons, *p* values were calculated using the Kruskal–Wallis rank sum test or Fisher’s exact test for continuous and categorical variables, respectively (Tables 1 and 2).

**TABLE 1:** Baseline demographics of MBC cohort.

	Total *N* = 1101 (%)

Median age	66.0
De novo	386 (35.1%)
Recurrent	715 (64.9%)
Race^∗^	
White	985 (89.5%)
Black	26 (2.4%)
American Indian/Alaska native	16 (1.5%)
Asian	61 (5.5%)
Pacific Islander	12 (1.1%)
Comorbidity score	
0	740 (67.1%)
1	178 (16.2%)
2	183 (16.7%)
ADI^∗^	
1–4	626 (56.9%)
5–7	309 (28.1%)
8–10	166 (15.0%)
RUCA^∗^	
Metropolitan	1060 (96.3%)
Rural	41 (3.7%)
Payor type	
Commercial	517 (47.0%)
Medicaid	46 (4.2%)
Medicare	389 (35.3%)
Multiple	149 (13.5%)
Clinic volume	
High	818 (74.3%)
Medium	76 (6.9%)
Low	207 (18.8%)

*Note:* RUCA = Rural Urban Commuting Area Codes.

Abbreviation: ADI, Area Deprivation Index.

^∗^Missing information was less than 0.1% and was included in the largest group.

**TABLE 2:** Biopsy at the time of recurrent MBC diagnosis.

	**No biopsy**	**Biopsy**	**Total**	**p** **value**

*N* (%)	361 (50.5%)	354 (49.5%)	715	
Median age	63.0	63.0	63.0	0.907
Comorbidity score				0.026
0	255 (53.1%)	225 (46.9%)	480	
1	59 (50.9%)	57 (49.1%0	116	
2	47 (39.5%)	72 (60.5%)	119	
ADI				0.847
1–4	218 (50.3%)	215 (49.7%)	433	
5–7	96 (52.2%)	88 (47.8%)	184	
8–10	46 (48.4%)	49 (51.6%)	95	
RUCA				1.000
Metropolitan	350 (50.5%)	342 (49.5%)	693	
Rural	11 (50.0%)	11 (50.0%)	22	
Payor type				0.406
Commercial	202 (53.2%)	178 (46.8%)	380	
Medicaid	16 (53.3%)	14 (46.7%)	30	
Medicare	100 (47.2%)	112 (52.8%)	212	
Multiple	43 (46.2%)	50 (53.8%)	93	
Clinic volume				0.030
High	244 (48.1%)	263 (51.9%)	507	
Medium	21 (45.7%)	25 (54.3%)	46	
Low	96 (59.3%)	66 (40.7%)	162	
Treatment				< 0.001
ET alone	225 (62.7%)	134 (37.3%)	43	
CDKi + ET	11 (25.6%)	32 (74.4%)	118	
CT	22 (18.6%)	96 (81.4%)	359	
Missing	103 (52.8%)	92 (47.2%)	195	

*Note:* RUCA = Rural Urban Commuting Area Codes; CDKi = CDK4/6 inhibitors; CT = chemotherapy.

Abbreviations: ADI, Area Deprivation Index; ET, endocrine therapy.

## 3. Results

### 3.1. Demographics

**FIGURE 1:**
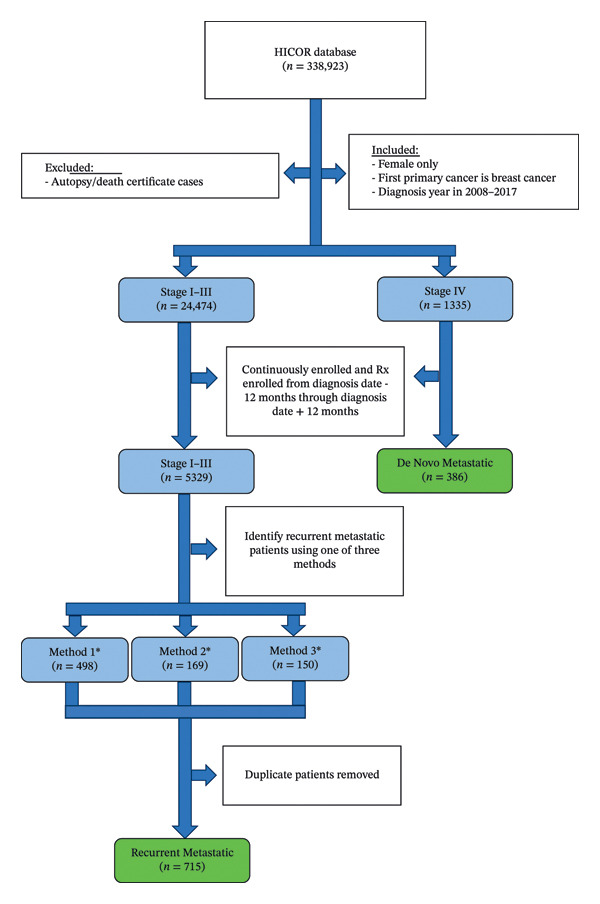
Cohort selection for metastatic breast cancer patients. ^∗^Methods definition is as follows: Method 1 identified patients who resumed systemic therapy within 4 months of the last breast cancer treatment (patients with breast cancer surgery within 1 year were removed to minimize capturing local recurrence). Method 2 identified patients who had ICD9/10 codes for metastatic disease with at least two claims on different dates (patients with secondary malignancy were removed to minimize capturing other metastatic cancers). Method 3 identified patients who had resumption of endocrine therapy within 18 months of the last breast cancer treatment.

We identified 1101 patients with MBC (715 patients with recurrent MBC and 386 patients with de novo MBC), as illustrated in Figure 1. Of the patients with recurrent or de novo MBC, patients were classified with ER+/HER2− MBC (485 recurrent MBC and 192 de novo MBC). Table 1 demonstrates the demographic profile of the MBC cohort. The median age at diagnosis of metastatic disease was 66 (range: 54–74). Over half of the cohort (52%) were older than 65 years. Among patients with MBC, the majority were Caucasian (89%), with the rest composed of Asian, Black, American Indian, and Hispanic patients. Based on the RUCA classification, patients predominantly lived in metropolitan neighborhoods (96%). Approximately 15% of patients lived in areas of high deprivation (ADI: 8–10). Patients had either Commercial (47%), Medicaid (4%), Medicare (35%), or multiple (13%) insurance. Most patients were treated at high‐volume centers (74%), though 19% of patients were seen at low‐volume centers.

### 3.2. Biopsy and Biomarker Testing in Recurrent MBC

**FIGURE 2:**
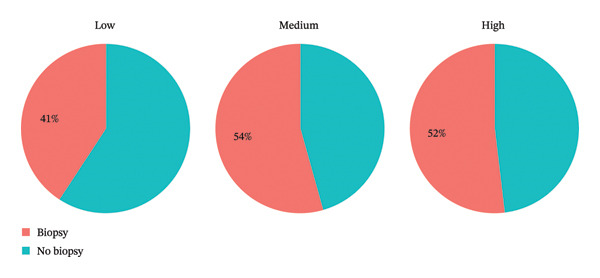
Receipt of biopsy at recurrence based on institution of care. Low‐volume centers defined as those that see < 25 patients with breast cancer/year, medium‐volume centers defined as those that care for 26–99 patients with breast cancer/year, and high‐volume centers defined as those that care for > 100 patients with breast cancer/year.

Of the patients with recurrent MBC (*n* = 715), 49.5% underwent a biopsy to confirm metastatic diagnosis. Similarly, 48.7% of recurrent MBC patients underwent biomarker reassessment. Patients with the highest comorbidity index (2) were more likely to undergo biopsy confirmation (60.5% vs. 39.5%, *p* = 0.02), as shown in Table 2. Patients treated at high‐ and medium‐volume centers had higher rates of biopsy than low‐volume clinics (51.9%, 54.3%, and 40.7%, respectively, *p* = 0.03), as shown in Figure 2. First‐line treatment selection was directly associated with the performance of biopsy and biomarker testing. ET alone was more common in patients who did not undergo biopsy (62.7% vs. 37.3%, *p* < 0.001) or biomarker reassessment (64.1% vs. 35.9%, *p* < 0.001).

### 3.3. First‐Line Treatment Selection in ER+/HER2− Cohort

**FIGURE 3:**
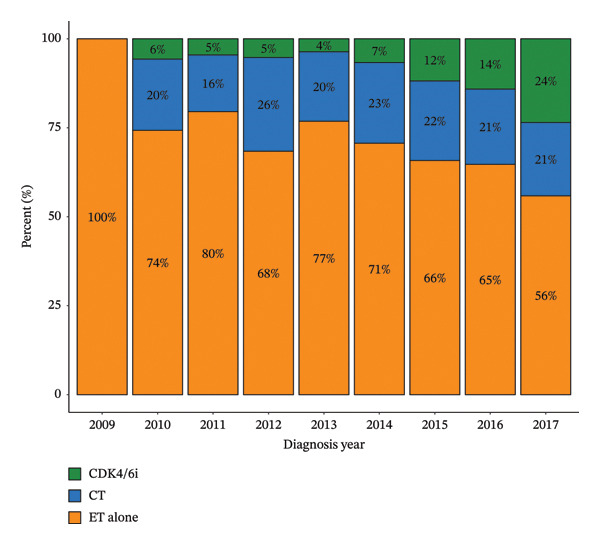
First‐line treatment for patients with ER+/HER2− MBC from 2009 to 2017 in WA state.

Of the patients with ER+/HER2− MBC (*n* = 677), first‐line treatment data within 6 months of diagnosis were available for a total of 527 patients. The majority of patients received ET alone (69%), followed by CT (22%) and CDKi + ET (9%). Temporal trends in treatment demonstrate increasing use of CDKi after the approval of the first in class, palbociclib in 2015, as shown in Figure 3 [22]. A high percentage of patients treated with CT as a first line received a combination of CT compared to a single agent (49% vs 51%). In the de novo MBC setting, first‐line CT (43%) receipt was similar to ET alone (48%, *p* < 0.001). Most patients who received CDKi + ET were < 65 years old (65.2%, *p* < 0.02). There was a trend toward less CT or CDKi + ET receipt in patients with the highest ADI, 8–10 (*p* = 0.88). In our study, none of the patients living in a rural setting (per RUCA classification) received CDKi + ET. We noted a trend that the majority of patients treated with CDKi + ET had commercial insurance compared to Medicare/Medicaid (60.9% vs. 26.1%, *p* = 0.10). Conversely, ET alone was primary treatment in patients with Medicare/Medicaid insurance (75.6%, *p* = 0.10). Almost all patients treated with CDKi + ET received care in a high‐volume center (91.3%, *p* = 0.11). Compared to patients with recurrent MBC, patients with de novo MBC were significantly more likely to receive CT (43.1% vs. 13.4%, *p* < 0.001) and less likely to receive ET alone (47.9% vs. 78.0%, *p* < 0.001). In the de novo MBC cohort, 17.3% received dual‐agent CT and 3.6% received > 2 agents.

### 3.4. Cost Estimates

Total costs (defined as costs from inpatient and outpatient claims 1 year after diagnosis) were estimated for patients and payors. Mean costs were further stratified by type of first‐line therapy: CDKi + ET ($15,216 and $116,125), CT ($17,294 and $145,170), and ET alone ($7646 and $35,875) for patients and payors, respectively (costs inflated to December 2019).

## 4. Discussion

Our study demonstrates that practice patterns for diagnosis and management of MBC vary across Washington state. Approximately half of the patients in this cohort did not undergo biopsy or biomarker reassessment at recurrence, despite their inclusion in guideline‐based diagnostic pathways. Patients treated in medium‐ and low‐volume centers were significantly less likely to undergo biopsy at the time of recurrence compared to high‐volume centers. Providers in these centers also demonstrate a trend toward treating MBC initially with dual‐agent CT or AI monotherapy, which represents a potential underutilization of CDKi in the early years of CDKi approval.

Several factors may contribute to the low biopsy rate at lower‐volume centers in WA. One barrier may be that approximately 15% percent of metastatic recurrences in the breast occur as bone‐dominant disease [23, 24]. Bone biopsy can present technical challenges: the procedure itself requires an IR suite with experienced providers, and proper specimen handling for accurate assessment of receptor status requires pathologists and technicians well‐versed in breast cancer. These resources are not available in all centers across the state; indeed, our institution has recently conducted a pilot study to evaluate the feasibility of providing good‐quality bone biopsy to patients from rural regions within our catchment area who have new suspected osseous recurrence of their breast cancer.

Second, community providers may not recognize the rationale for obtaining a new biopsy, perhaps because they overestimate the concordance of primary breast pathology and recurrence site, or because they place confidence in commercially available liquid biopsies to confirm disease and guide therapy. While liquid biopsy holds promise in assessing early recurrence in MBC, it does not allow histopathological confirmation specifically of breast cancer, does not provide receptor information, and is limited by lower sensitivity than tissue biopsy [25–27]. Misconceptions around these issues, if present, pose risks for patient care, and more research is needed to clarify provider‐ and institution‐level decision‐making around diagnostic practices for MBC across the state.

Once a diagnosis of MBC was established in members of our cohort, either by biopsy or presumption, endocrine monotherapy alone was the most administered first‐line treatment. Although this was historically the standard of care, CDK4/6i‐based therapy has supplanted endocrine monotherapy as a recommended first‐line therapy of ER+/HER2−MBC in the modern era [12]. Forty percent of the patients in the database were treated in the pre‐CDK4/6i era (prior to 2015), which may suggest underestimation of CDK4/6i utilization. It is reassuring that there is an increasing trend in CDK4/6i utilization annually from 7% in 2014 up to 24% by 2017, which later evaluations of our data may reflect.

Twenty‐two percent of patients in our cohort received first‐line CT, with CT offered significantly more frequently in low‐ and middle‐volume centers. These data confirm, in an MBC context, observations made by Ramsey et al. demonstrating that adherence to the ASCO/ABIM Choosing Wisely measures in cancer varies widely in Washington State [28]. CT utilization was also notably high in patients with de novo ER+/HER2− MBC, which may reflect a trend to treat all “new” patients regardless of stage with combination CT. This finding is surprising considering that patients naïve to estrogen‐based therapy should be particularly sensitive to endocrine‐based therapies. Admittedly, data from the RIGHT CHOICE trial, which demonstrated the effectiveness of CDK4/6i over CT as first line in patients with aggressive disease, were not available at the time of treatment selection for these patients [29].

Approximately 20% of patients with de novo MBC receiving CT in our cohort were given polychemotherapy, with 17% given a dual‐agent regimen. This diverges from both NCCN guidelines and ASCO Choosing Wisely. Despite evidence suggesting limited incremental benefit from combination CT in metastatic disease, nearly one‐fifth of de novo patients in our cohort received multiagent regimens, raising concerns about treatment burden [30–32]. A rare exception can be made in cases of imminent need for rapid response to relieve tumor‐related symptoms, but few patients with MBC present with such clinical emergencies [12].

We explored potential factors that may influence MBC treatment decisions in WA. Age significantly correlated with treatment selection, with patients > 65 being less likely to receive CDK4/6i‐based therapy. This is noteworthy, as emerging data suggest that CDKi benefit elderly women with MBC. Real‐world data presented by Rugo demonstrated improved progression‐free survival and overall survival on CDK4/6i (palbociclib)‐based therapy among older patients (> 65) [33]. Data from the United Kingdom show high clinical benefit rates for women > 75 years old treated with palbociclib and ET [34].

Our data cannot provide insight into disparities between patients with and without insurance, a well‐established factor for patient outcomes [35]. Our data do, however, highlight that type of insurance and location of care are associated with treatment selection. Most patients treated with CDK4/6i carried commercial insurance rather than Medicare/Medicaid, and almost all patients treated with CDK4/6i received care at a high‐volume center. Our data did not show differences in treatment selection based on race or socioeconomic status, which are also strongly associated with breast cancer mortality [36]. We suspect that these factors could not be fully captured, as our eligibility criteria necessitated a continuous enrollment period for insurance, limiting the inclusion of a broader group of patients. Together, these findings suggest that access to effective medications and the costs of co‐pays may be a driving factor for disparities in MBC.

We assessed the total costs for patients and payors to understand potential financial pressures influencing treatment selection. As expected, endocrine monotherapy is the lowest cost option for both patients and payors. Surprisingly, however, CT was most expensive to both patients and payors. Although many CT agents in breast cancer are old and relatively inexpensive drugs, infusion and administration costs remain substantial [37]. High co‐pays for targeted therapies, including CDKi, have often been cited as barriers to access for patients with less financial support, resulting in overuse of CT in this vulnerable patient population [38]. Our cost analysis indicates that, when administration and supportive care are included, CT can represent a substantial financial burden for both patients and payors.

Differences in diagnostic infrastructure and access to subspecialty pathology services at lower‐volume centers may partially explain the lower biopsy rates observed in our study [39–41]. Chal suggests that practices diverging from nationally accepted guidelines may play a role in this discrepancy. Potential solutions to improve adherence could include referral to high‐volume centers for biopsy and biomarker reassessment, as well as alternatives/adjuncts to biopsy. Novel imaging techniques, including 16‐alpha‐^18^F‐fluoro‐17 estradiol (FES)–PET, primarily available in higher‐resource tertiary settings, may also aid with diagnosis [42, 43].

More research is needed to determine drivers of guideline nonadherence in lower‐volume centers. One can hypothesize, however, that pressure to provide increasingly complex cancer care across a broad spectrum of disease types with sparse resources and staff may be contributing. If so, collaborative ties to specialists in tertiary centers could help community providers remain currently in practice, while also allowing patients to benefit from clinical trials and opportunities for second opinions at large urban academic institutions.

Our study has several limitations, many of which stem from the nature of the dataset. Though the HICOR database provides a novel linkage between high‐quality cancer registry data and insurance claims from major providers across Washington, it does not have electronic health record (EHR) data; thus, we have a limited window into the clinical rationale behind practice patterns. Our cohort may underestimate the MBC population (late recurrences can occur > 10–15 years) and be biased toward patients receiving treatment [44]. Our sample size is large enough to capture most recurrences, and our cohort reflects the real world because the majority of patients receive at least first‐line treatment. Improper claim code inputs may result in missed cases, but this is uncommon. We performed several quality metrics to ensure comprehensive code selection. For example, to ensure that we had the appropriate codes for identification of our recurrent MBC cohort, we tested the codes for biopsy and biomarker testing in our newly diagnosed MBC cohort (all of whom should have this testing as standard of care). Finally, gaps in insurance coverage and clinical care cannot be assessed with the HICOR database, and patients may receive care beyond what is documented. We attempted to account for this in the study eligibility criteria, requiring continuous enrollment for a defined period.

## 5. Conclusion

Our study identifies specific targets for intervention, including diagnostic practices and early treatment selection, that may inform future quality‐improvement efforts. Next steps will include data‐driven educational outreach projects, benchmarking, development of tracers to enhance molecular staging of breast cancer, and study of the efficacy of initial therapies in community settings. Our study demonstrates the need for initiatives to standardize quality of care relative to clinical guidelines in MBC care.

## Author Contributions

Poorni M. Manohar: conceptualization, methodology, formal analysis, investigation, data curation, writing–original draft, writing–review and editing, visualization, project administration, and funding acquisition.

Veena Shankaran: methodology, formal analysis, data curation, writing–review and editing, and supervision.

Nancy E. Davidson: writing–review and editing and supervision.

Natasha Hunter, William R. Gwin, Rachel L. Yung, and Jennifer M. Specht: writing–review and editing.

Catherine Federenko: conceptualization, methodology, formal analysis, software, investigation, data curation, writing–review and editing, and project administration.

Sun Qin, Qian (Vicky) Wu, and Jenna M. Voustinas: conceptualization, methodology, software, formal analysis, investigation, and data curation.

Josh A. Roth: conceptualization, methodology, formal analysis, investigation, data curation, writing–review and editing, visualization, project administration, and funding acquisition.

Hannah M. Linden: conceptualization, methodology, formal analysis, investigation, data curation, writing–review and editing, visualization, project administration, and funding acquisition.

## Funding

This study was supported by the National Institutes of Health (T32CA009515), the American Society of Clinical Oncology (ASCO YIA Grant), and Pfizer (Pfizer Competitive Grants Award).

## Conflicts of Interest

The authors declare no conflicts of interest.

## Data Availability

The data that support the findings of this study are available from HICOR. Restrictions apply to the availability of these data, which were used under license for this study. Data are available from the authors with the permission of the Hutchinson Institute for Cancer Outcomes Research.
